# Supplementary material for: How common depictions of wealth distributions can bias people to underestimate inequality
Source: Nat Commun. 2026 Mar 6;17:3897. doi: 10.1038/s41467-025-62422-5 (PMC13125609; doi:10.1038/s41467-025-62422-5)
Supplement: Supplementary file 1 — Supplementary Information [file 41467_2025_62422_MOESM1_ESM.pdf]

Supplementary Materials for

How common depictions of wealth distributions can bias people to underestimate inequality

This file includes:

- Pilot study methods and results
- Additional methodological detail for each study
- Figures S1 – S7
- Tables S1 – S14

Supplementary Methods

All preregistrations, survey materials, data, and code are available on ResearchBox: https://researchbox.org/380

Pilot Study: Misperceptions of Inequality
In a pilot study, we examine misunderstandings and misperceptions when people evaluate economic distributions. We find that this is a deceptively difficult task for most people. We conducted an experiment to identify the specific errors that people make and how these errors influence people's opinions of national economic distributions. Further, we test to see if the training module we designed significantly reduces the errors that people make when making judgments and decisions about such distributions.

1.1 Methods and Participants. All research conducted for this project complied with all relevant ethical regulations and was approved by the Institutional Review Board at the University of California, Los Angeles. In the Pilot study included with Study 2, we recruited 251 participants from Amazon's Mechanical Turk who passed pre-registered inclusion criteria of passing an attention check and spending at least 6 minutes on the full survey (51% female, M_{age} = 38.4, SD_{age} = 12.6). Participants were randomly assigned between-subjects to one of two conditions: Training or No Training. Participants in the Training condition completed a seven-minute interactive training module designed to introduce the core concepts of wealth distributions: Population percentiles, allocation of a fixed percentage of a resource, calculating average allocation per percentile, and monotonic allocations. This Training module contained definitions, descriptions and examples, and it had interactive questions where participants answered a question and then were shown a correct answer. At the end of the training module, participants responded to three comprehension questions (e.g., "If you gave 30% of total wealth to the richest 10% of people in the United States, how much wealth is remaining to be distributed to the rest of the population?"). After this, participants completed the Ideal Allocation Task, constructing their ideal allocation of wealth across five population quintiles, and the Descriptive

Allocation Task, constructing what they believed to be the *current* distribution of wealth across the five quintiles (tasks based on Norton and Ariely, 2011). Participants in the No Training condition first completed the Ideal and the Descriptive Allocation Task, and then answered the comprehension questions. The order of the Ideal and Descriptive Allocation Tasks was randomized between participants.

We captured three measures of miscomprehension: errors on the comprehension questions, failure to allocate 100% of national wealth across population groups, and violations of monotonicity (allocating of a larger share of wealth to a definitionally poorer group). For instance, the following allocation represents the actual response of a participant who violated monotonicity in the Ideal Allocation Task.

Table S1. Example Response

Example of a participant response in the Pilot Study that violated monotonicity

Population Group	Percent of National Wealth they SHOULD Control
**Richest 20%**	*50%*
**Next 20%**	*10%*
**Next 20%**	*10%*
**Next 20%**	*10%*
**Poorest 20%**	*20%*
**Total**	100%

As can be seen, this participant suggested that the poorest quintile of Americans should hold twice as much wealth as the next-richest quintile of Americans. We were interested in how pervasive these sorts of errors were and, secondarily, whether training helped to mitigate them.

Norton and Ariely (2011) found that an unreported number of participants in their study violated monotonicity (the authors simply corrected this mistake for participants by rearranging allocation amounts monotonically) and others failed to allocate exactly 100% of national wealth (the authors rescaled participants' responses for them). While, in this study and in all subsequent ones using this paradigm, we made sure that this latter error would not result from a simple arithmetic error by displaying a live-updating sum of the share of wealth participants had allocated, we were curious whether these mistakes were in fact indicative of a deeper misunderstanding rather than a simple mistake.

1.2 Results. We find evidence for pervasive misunderstandings of economic distributions. Without training, 75% of participants made at least one error and 27% made the fundamental error of violating monotonicity. Figure 1 shows the number of people remaining in the sample after removing participants who made each specific error type (failed to sum to 100% of national wealth, violated monotonicity, and answered the comprehension questions correctly).

Figure S1. Pilot Study errors in the condition where participants did not receive training on understanding economic distributions

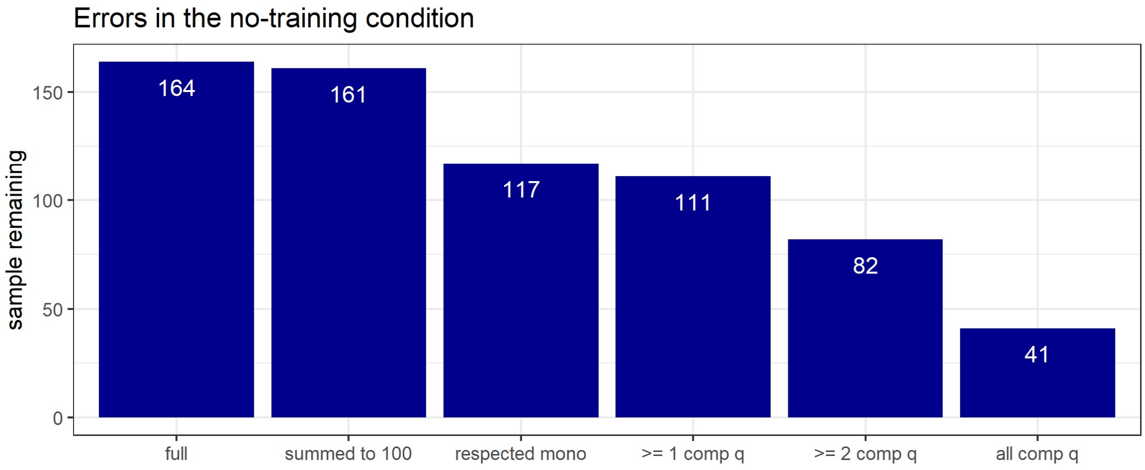

Notes: Displaying resultant sample after removing participants who failed each respective measure of comprehension in the Ideal Allocation Task.

While the training module successfully reduced violations of monotonicity from 27% to 15% ($\chi^2(1,251) = 3.92$, $p < .05$), these results show that full comprehension of the task seems to be deceptively hard for a large share of people.

2. Study 1: Unit Dependence

The purpose of Study 1 was to demonstrate unit-dependence, that people's judgments of the fairness of unevenly partitioned population groups would be influenced by the metrics used to express economic inequality. We pre-registered a prediction that people would perceive distributions to be less unfair and they would have lower support for redistributional policies when inequality was expressed using the (commonplace) metric of Total Wealth compared to a metric that scales by population size (i.e., average wealth).

2.1 Methods and Participants. In Study 1, we recruited 400 participants from Amazon's Mechanical Turk, analyzing results of 391 participants who met pre-registered inclusion criteria (55% female, $M_{age} = 38.8$, $SD_{age} = 12.8$). Participants were asked their opinions about how wealth is distributed in an unnamed "Western, democratic" country (the information we provided approximated the current wealth distribution in the United States, but participants were not informed of this). Participants were then randomly assigned to either a Total Wealth or Average Wealth condition in which the same distribution of wealth was presented in different formats. In the Total Wealth condition, participants were presented with the wealth held by the richest 10% and poorest 90% of people using a metric of total national wealth. In the Average Wealth

condition, this information was presented using a metric of average wealth per percentile (Figures 1A and 1B).

Participants then responded to two questions: “In your opinion, how fair is this distribution?” (-5=completely unfair, 0=neither fair nor unfair, 5=completely fair), and “Given this distribution, to what extent would you support a government policy that redistributes wealth from richer to poorer individuals?” (-5=strongly oppose, 0=neither oppose nor support, 5=strong support). We pre-registered a prediction that people would rate the economic distribution as less fair and indicate greater support for redistribution when the same information is displayed in the Average Wealth condition compared to those in the Total Wealth condition.

2.2 Results. As predicted, we find that support for redistribution was significantly greater ($b=1.40$, $t=3.39$, $p<.001$) in the Average Wealth condition. This condition uses a “partition-invariant metric” that scales the wealth allocation by the size of the group, leading to policy judgments that are not biased by arbitrary differences in the size of the identified population groups. Importantly, we observed marginal effects on perceived fairness of the distribution, despite predicting that those in the Average Wealth condition would see the distribution to be significantly less fair ($b=-.52$, $t=-1.78$, $p=.076$). However, when we perform subset analyses among only participants who, after the main DVs, correctly recalled the unit in which the distributional information was displayed, the differences between conditions were significant ($b=-.79$, $t(262)=-2.34$, $p=.020$).

3. Study 2: Partition-Dependence

In Study 2, we test the first hypothesis of the *salient-group inequality (SGI) heuristic*, that people are under-sensitive to the size of different groups in judgments of economic inequality. We find evidence in support of the hypothesis that judgments are biased according to the arbitrary partitioning of the population groups.

We note that here, as with all studies regarding Partition Dependence (i.e., Studies 2 – 5) which use the paradigm slightly adapted from Norton and Ariely (2011), we include the training module at the start of the study. Noting that this module had a significant effect on participant understanding of the task, we wanted to increase the reliability of participants’ preferences expressed in these studies. In other words, we wanted to maximize the chances that participants were giving meaningful responses. Thus, when we document effects of Partition Dependence, we take our estimates to be a lower-bound measure of the size of the effect absent this training.

3.1 Methods and Participants. In Study 2, we recruited 200 participants from Amazon’s Mechanical Turk, analyzing results of 165 participants who met pre-registered inclusion criteria (44% female, $M_{age} = 38.0$, $SD_{age} = 9.5$). Participants were randomly assigned to construct the actual and ideal wealth distribution of the United States using one of two population partitions.

In the Quintiles condition, participants used population quintiles (e.g. richest 20% of Americans, next 20%,..., poorest 20%), and in the Logarithmic condition the population groups were defined as richest 1%, next 4%, next 15%, next 30%, poorest 50% (Figures 2A and 2B). In each condition, participants indicated what they believed to be the *current* wealth distribution in the United States (Descriptive Allocation Task) as well as their opinion on how wealth ideally should be distributed (Ideal Allocation Task). Afterwards, participants answered a few demographic questions including partisanship (using a 7-point scale of likelihood of voting Republican on a generic ballot question), fiscal conservatism (When it comes to your attitudes on fiscal issues, how would you rate yourself on the following scale? 1=Extremely conservative, 7=Extremely liberal), MacArthur Subjective Social Status Scale (1-10), and self-placement on an item from the World Values Survey: 1 = In the long run, hard work usually brings a better life, 10 = Hard work doesn't generally bring success—it's more a matter of luck and connections

3.2 Results. As predicted, participants in the Logarithmic condition allocated significantly more wealth to the richest 20% in both the Ideal and Descriptive Allocation task, as compared to participants in the Quintiles condition (Table S1). Thus we find further evidence that judgments of economic inequality are partition-dependent such that people are under-sensitive to the size the of the population groups. People allocate more wealth to the richest 20% of Americans when this population is partitioned into four (versus one) group. This pattern is consistent with the SGI heuristic.

Table S2: Study 2 effects of partitioning the population Logarithmically versus by Quintiles on descriptive and ideal wealth allocations

	Dependent variable:			
	Descriptive		Ideal	
	(1)	(2)	(3)	(4)
Quintiles Condition	-28.510***	-26.345***	-21.457***	-22.204***
	(3.844)	(3.517)	(3.082)	(2.992)
	p = 0.000	p = 0.000	p = 0.000	p = 0.000
Republicanism		-0.971		0.527
		(0.937)		(0.797)
		p = 0.302		p = 0.510
Fiscal Conservatism		1.634		-0.736
		(1.145)		(0.974)
		p = 0.156		p = 0.451
Sucess External		-1.863**		-1.320*
		(0.698)		(0.594)
		p = 0.009		p = 0.028
MSSSS		-4.789***		2.888***
		(0.973)		(0.828)
		p = 0.00001		p = 0.001
HH Income		1.236		-0.450
		(0.704)		(0.599)
		p = 0.082		p = 0.455
Constant	73.510***	97.777***	48.494***	44.958***
	(2.710)	(9.660)	(2.173)	(8.217)
	p = 0.000	p = 0.000	p = 0.000	p = 0.00000
Observations	165	165	165	165
R^2	0.252	0.422	0.229	0.330
Adjusted R^2	0.248	0.400	0.224	0.304
Residual Std. Error	24.690 (df = 163)	22.044 (df = 158)	19.796 (df = 163)	18.751 (df = 158)
F Statistic	54.996*** (df = 1; 163)	19.246*** (df = 6; 158)	48.465*** (df = 1; 163)	12.945*** (df = 6; 158)

Note: *p<.05, **p<.01, ***p<0.001

Here we report unstandardized coefficients and standard errors from OLS regression models (two-sided tests, no multiple-hypothesis corrections). Models 1 and 2 report the effects on total allocation of wealth to the richest 20% of Americans in the Descriptive Allocation Task (participants' estimates of the true distribution of wealth in the United States). Models 3 and 4 report the effects on total allocation of wealth allocated to the richest 20% of Americans in the Ideal Allocation Task (participants report what the wealth distribution of the United States ideally should be, in their opinion).

The effects of condition hold controlling for the battery of political opinions. As can be seen, for both the Descriptive and Ideal condition, in both the controlled and uncontrolled regressions, considerably more wealth was allocated to the top 20% of Americans when they represented more population groups.

4. Study 3: Different Number of Groups

In the previous study, we found a large partitioning effect when the richest 20% of the population was partitioned into three sub-groups (richest 1%, next 4%, next 15%). In Study 3, we test the generalizability of partition dependence in judgments of economic distributions using an alternative method of dividing national wealth. We wanted to ensure that the effects of partitioning were not unique to an uneven, quasi-logarithmic unpacking of the top wealth group. Thus, in Study 3, we unpacked the top wealth group into evenly spaced population groups.

4.1 Methods and Participants. In Study 3, we recruited 250 participants from Amazon's Mechanical Turk, analyzing results of 162 participants who met pre-registered inclusion criteria (51% female, M_{age} = 37.7, SD_{age} = 11.8). Following the same paradigm as in Study 2, participants completed the Descriptive and Ideal Allocation Tasks using one of two population partitions. In the {50-50} condition, participants simply estimated how much wealth was held by the "richest 50% of Americans" and the "poorest 50% of Americans," as well as how much wealth they thought each of these groups ideally should hold. In the {10-10-10-10-10-50} condition, the richest 50% of Americans were partitioned into 5 deciles.

4.2 Results. As predicted, people allocated significantly more wealth to the richest 50% of Americans in both the Ideal and Descriptive Allocation Task when this population group was partitioned into five deciles.

Table S3: Study 3 effects of a 50-50 partition versus a 10-10-10-10-10-50 partition on descriptive and ideal wealth allocations

	Dependent variable:			
	Allocation to Top 20%: Descriptive		Allocation to Top 20%: Ideal	
	(1)	(2)	(3)	(4)
unpacked condition	8.029	10.760	7.635	9.188
	t = 4.793	t = 5.988	t = 3.314	t = 3.232
	p = 0.00001	p = 0.00000	p = 0.002	p = 0.002
republicanism		-0.419		-3.194
		t = -0.135		t = -0.647
		p = 0.894		p = 0.519
fiscal conservatism		0.159		-1.541
		t = 0.214		t = -1.307
		p = 0.832		p = 0.195
WVS: Incentivize		-0.535		0.993
		t = -1.025		t = 1.202
		p = 0.309		p = 0.233
WVS: Success as Luck		0.330		-0.146
		t = 0.821		t = -0.229
		p = 0.414		p = 0.820
MacArthur SES		-1.338		0.553
		t = -2.283		t = 0.596
		p = 0.025		p = 0.553
Constant	87.801	95.122	64.570	67.681
	t = 104.841	t = 17.208	t = 56.044	t = 7.740
	p = 0.000	p = 0.000	p = 0.000	p = 0.000
Observations	162	104	162	104
R^2	0.126	0.327	0.064	0.182
Adjusted R^2	0.120	0.285	0.058	0.132
Residual Std. Error	10.607 (df = 160)	8.961 (df = 97)	14.593 (df = 160)	14.176 (df = 97)
F Statistic	22.976*** (df = 1; 160)	7.853*** (df = 6; 97)	10.979** (df = 1; 160)	3.604** (df = 6; 97)

Notes:

Here we report unstandardized coefficients and standard errors from OLS regression models (two-sided tests, no multiple-hypothesis corrections). Models 1 and 2 report the effects on total allocation of wealth to the richest 20% of Americans in the Descriptive Allocation Task (participants' estimates of the true distribution of wealth in the United States). Models 3 and 4 report the effects on total allocation of wealth allocated to the richest 20% of Americans in the Ideal Allocation Task (participants report what the wealth distribution of the United States ideally should be, in their opinion).

These findings indicate that people are willing to tolerate more wealth inequality when the richest segment of the population is subdivided into more groups. Consistent with the SGI heuristic, we find a bias toward even allocation between groups without sufficient sensitivity to differences in group sizes.

5. Study 4: Actual Distribution Given

In Study 4, we focused on the Ideal Allocation Task only. In Studies 2 and 3, we found that participants' estimates of the actual wealth distribution of the United States were systematically biased by the arbitrary partitioning of population groups. These inaccurate estimates of the actual wealth distribution may partially explain people's opinions about their preferred distribution. Therefore, in Study 4, we tested whether partitioning effects on *preferred* economic inequality hold when people have accurate information about the current distribution.

5.1 Methods and Participants. In Study 4, we targeted 500 participants and ended up recruiting 507 participants from Amazon's Mechanical Turk, analyzing results of 475 participants who met pre-registered inclusion criteria (44% female, M_{age} = 41.5, SD_{age} = 13.2). Participants were presented with accurate information on the current wealth distribution in the United States (using a 50-50 population partition). Then, participants were randomly assigned to either a Deciles condition or Quartiles condition to indicate their ideal wealth distribution (using the same Ideal Allocation Task as in Studies 2 and 3). In the Deciles condition, the population was partitioned into six groups (richest 10%, next 10% next 10%, next 10%, next 10%, poorest 50%). In the Quartiles condition the population was partitioned into three groups (richest 25%, next 25%, poorest 50%).

5.2 Results. Despite having accurate information regarding the way wealth is currently allocated, people's ideal constructions of wealth distributions would remain partition dependent. This is, in fact, what we find. Controlling for the political covariates described in Study 2 and Study 3, we find that participants in the Deciles condition allocated 72% of national wealth to the top half of the distribution whereas participants in the Quartiles condition allocated just 64% ($b=7.99$, $t=2.78$, $p < 0.01$). This result is directionally similar to the uncontrolled regression not accounting for political differences, but this uncontrolled regression is only marginally significant ($b=5.31$, $t=1.73$, $p = 0.086$).

6. Study 5: Partition-Invariant Metrics for Displaying Economic Distributions

The previous studies demonstrate that judgments of economic inequality are under-sensitive to differences in the size of population groups presented. This results in a systematic bias when economic distributions are presented with population groups of unequal size, which is typical in the popular press (e.g., comparing the richest 1% of Americans versus the bottom 99%).

Journalists and policymakers use these types of comparisons to highlight the extent of the wealth gap, but doing so leads readers and voters to form biased impressions—systemically underestimating economic inequality and the need for policies to redistribute wealth. In Study 5, we tested a method to reduce this bias using metrics of displaying economic inequality that are *partition-invariant*.

6.1 Methods and Participants. In Study 5, we recruited 800 participants from Amazon's Mechanical Turk, analyzing results of 679 participants who met pre-registered inclusion criteria (56% female, M_{age} = 37.7, SD_{age} = 12.0). Participants were randomly assigned to one of four experimental conditions in a 2 (partitioning: quintiles versus logarithmic) x 2 (units: total versus average wealth) experimental design. The first experimental factor varied whether participants were presented with population groups partitioned either into quintiles (richest 20%, next 20%, …, poorest 20%) or quasi-logarithmically (richest 1%, next 4%, next 5%, next 10%, next 20%, …, poorest 20%). The second experimental factor varied the metric used to define the amount of wealth held by each population group. Half of participants were presented with the distribution using a Total Wealth metric (i.e., displaying the percentage of total wealth held by a given population group), and the other half of participants were presented with an Average Wealth metric (i.e., the average wealth held per percentile; percent-per-percentile). The latter metric is partition invariant since it is scaled by the number of individuals in a group.

Participants observed the wealth distribution and then responded to three questions asking their opinions on the fairness of the distribution and three questions asking about the extent to which they would support redistributing wealth from richer to poorer citizens. The order of the three questions within each block was randomized. Participants were shown a given distribution and asked to place themselves on the following scales:

- $Fairness_a$: 1=Ideally, rich Americans would own much more wealth, 6=Ideally, no change from this distribution, 11=Ideally, poor Americans would own much more wealth
- $Fairness_b$: 1=Ideally, the wealth distribution should be much more unequal, 6=Ideally, no change from this distribution, 11=Ideally, the wealth distribution should be much more equal
- $Fairness_c$: 1=This distribution is not at all unfair, 7=This distribution is extremely unfair
- Policy $Support_a$: 1=Given this distribution, I would strongly oppose a government policy that redistributes more money to the poor, 6=Neither oppose nor support, 11=Given this distribution, I would strongly support a government policy that redistributes more money to the poor
- Policy $Support_b$: 1= Given this distribution, I think the government should significantly decrease taxes on the rich, 6=Taxes on the rich should stay the same as they are today, 11=Given this distribution, I think the government should significantly increase taxes on the rich
- Policy $Support_c$: 1=Given this distribution, I think the government should significantly decrease services that benefit the poor, 6=Government services that benefit the poor should stay the same as they are today, 11=Given this distribution, I think the government should significantly increase services that benefit the poor

From these six questions we constructed composite measures of fairness (α=0.70) and support for redistribution (α=88).

We pre-registered a prediction that the partition dependence bias would we be diminished (i.e. a smaller difference between the alternative population partitions in terms of reported fairness and support for redistributions) when wealth is expressed using a partition-invariant metric (i.e., average wealth).

6.2 Results. As predicted, using the partition-invariant metric of "percent per percentile" (analogous to average wealth) reduced the difference between partition effects on fairness judgments and support for redistributional policy. We observe a significant interaction effect between our two treatment factors such that partitioning effects on fairness (b=1.49, t=3.56, p<.001) and support for redistribution (b=1.00, t=2.09, p<.05) are diminished when wealth is displayed using a partition-invariant metric (Figure S2 and S3).

Figure S2. Study 5 effects of metric and partitioning on judgments of unfairness

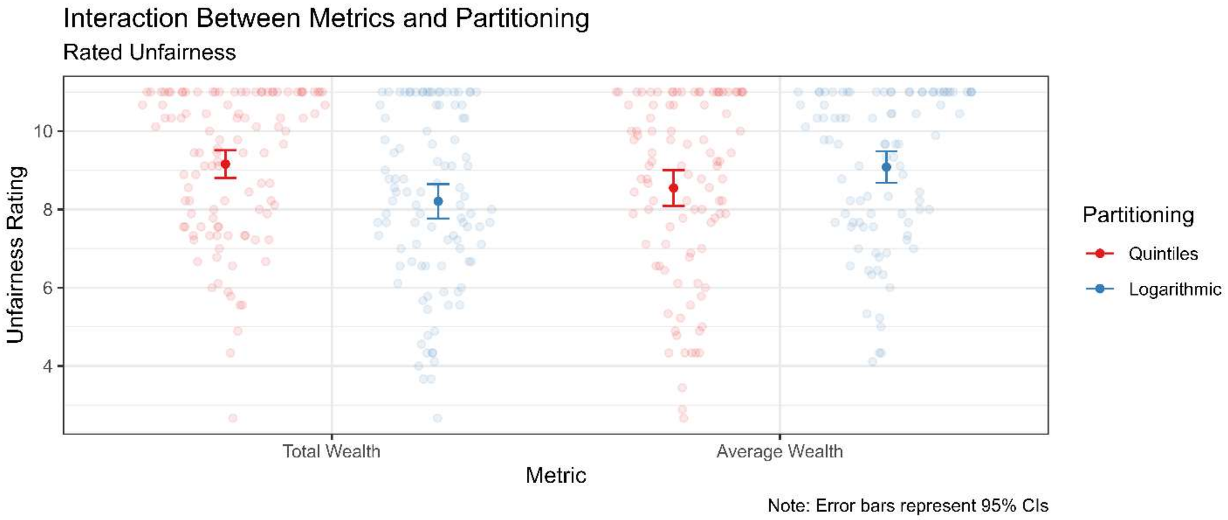

Note: Displaying means (dark points) and 95% Confidence Intervals for treatment effects on indexed judgments of unfairness. Each translucent point represents one participant's response (*N*=679).

Figure S3. Study 5 effects of metric and partitioning on support for redistribution

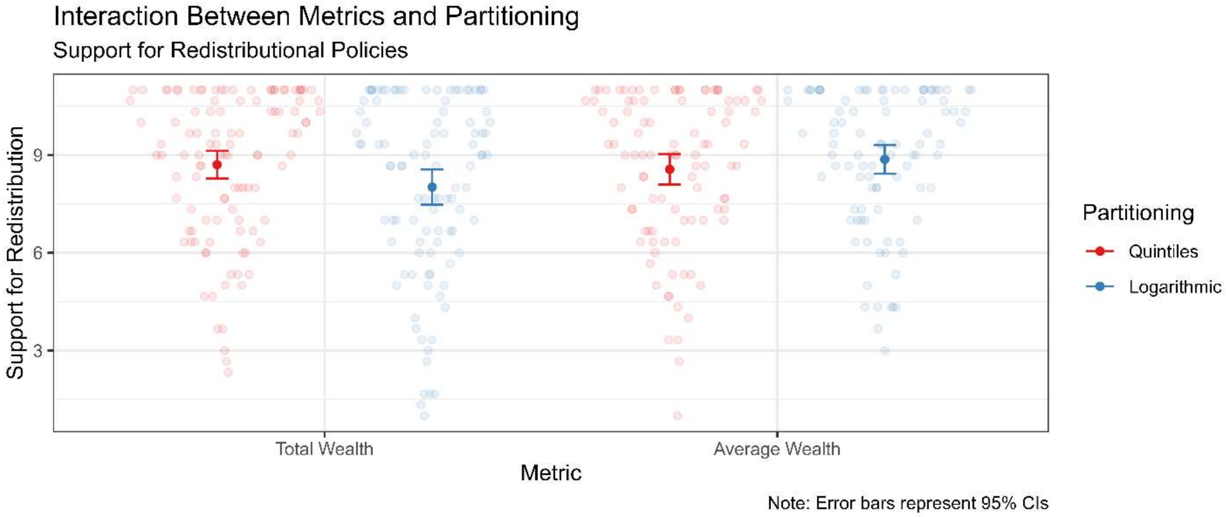

Note: Displaying means (dark points) and 95% Confidence Intervals for treatment effects on indexed judgments of support for redistributive policies. Each translucent point represents one participant's response (*N*=679).

Because the reliability of the fairness index was on the lower end of acceptability, we separately regressed each of the three measures in both a controlled and uncontrolled regression (as described above). In all cases, the results were substantively unchanged: The effect of partitioning was significantly smaller when the distribution was expressed using an Average Wealth metric.

Furthermore, we asked several other dependent measures (labels correspond to the following regression table):

1. **Unfairness**: 1-7 judgment of fairness (1=This distribution is not at all unfair, 7=This distribution is extremely unfair)
2. **Pref. More Equality**: 1-11 preference for inequality (1=Ideally, the wealth distribution should be much more unequal, 6=Ideally, no change from this distribution, 11=Ideally, the wealth distribution should be much more equal)
3. **Transfer Wealth to Less Wealthy:** 1-11 preference for ideal allocation (1=Ideally, rich Americans would own much more wealth, 6=Ideally, no change from this distribution, 11=Ideally, poor Americans would own much more wealth)
4. **Support Redistribution:** 1-11, preference for government redistribution (1=Given this distribution, I would strongly oppose with a government policy that redistributes more money to the poor, 6=Neither oppose nor support, 7=Given this distribution, I would strongly support with a government policy that redistributes more money to the poor)
5. **Take the Wealthy:** 1-11, preference for tax policy (1=Given this distribution, I think the government should significantly decrease taxes on the rich, 6=Taxes on the rich should

stay the same as they are today, 11=Given this distribution, I think the government should significantly increase taxes on the rich)

6. **Increase Safety Net:** 1-11, preference for social safety net (1=Given this distribution, I think the government should significantly decrease services that benefit the poor, 6=Government services that benefit the poor should stay the same as they are today, 11=Given this distribution, I think the government should significantly increase services that benefit the poor)

Results are presented in the following table.

Table S4. Regressions results from Study 5.

All Dependent Measures

	Dependent variable:					
	Unfairness	Pref. More Equality	Transfer Wealth to Poor	Support Redistribution	Tax the Rich	Increase Safety Net
	(1)	(2)	(3)	(4)	(5)	(6)
Log Condition	-0.852	-0.513	-1.489	-0.955	-0.583	-0.531
	t = -2.167	t = -1.800	t = -3.709	t = -2.180	t = -1.800	t = -1.693
	p = 0.031	p = 0.073	p = 0.0003	p = 0.030	p = 0.073	p = 0.092
Invariant Metric	-0.388	-0.584	-0.863	-0.084	-0.198	-0.150
	t = -0.981	t = -2.037	t = -2.139	t = -0.192	t = -0.609	t = -0.475
	p = 0.328	p = 0.043	p = 0.034	p = 0.849	p = 0.544	p = 0.636
Interaction	1.325	1.036	2.097	1.330	0.805	0.850
	t = 2.330	t = 2.512	t = 3.611	t = 2.099	t = 1.717	t = 1.871
	p = 0.021	p = 0.013	p = 0.0004	p = 0.037	p = 0.087	p = 0.063
Constant	8.997	9.523	8.954	8.064	9.138	8.917
	t = 33.006	t = 48.178	t = 32.166	t = 26.539	t = 40.668	t = 40.970
	p = 0.000	p = 0.000	p = 0.000	p = 0.000	p = 0.000	p = 0.000
Observations	402	402	402	402	402	402
R^2	0.017	0.016	0.039	0.021	0.011	0.013
Adjusted R^2	0.009	0.009	0.032	0.014	0.003	0.005
RSE (df = 398)	2.846	2.064	2.906	3.172	2.346	2.272
F (df = 3; 398)	2.267	2.161	5.371^{**}	2.837^{*}	1.442	1.707

Note: Here we report unstandardized coefficients and standard errors from OLS regression models (two-sided tests, no multiple-hypothesis corrections). Each model tests the extent to which the effect of quasi-logarithmic partitioning of the population ('Log Condition') depends on whether or not a partition-invariant metric--average wealth per group--was used ('Invariant Metric'). The focal test is of the interaction coefficient, 'Interaction'.

7. Study 6: Middle Neglect

In Study 6, we test the second prediction of the SGI heuristic: judgments of inequality under-weight wealth held by the middle of the economic distribution relative to the extremes. We posit that people focus on a comparison between the most salient groups—the richest and poorest population segments—and therefore judgments are relatively insensitive to changes in the middle of the distribution.

7.1 Methods and Participants. In Study 6, we recruited 200 participants from Amazon's Mechanical Turk, analyzing results of 197 participants who met pre-registered inclusion criteria (41% female, M_{age} = 34.6, SD_{age} = 11.4). Participants were asked to make a series of judgments about the economic distribution of eight anonymous Western, democratic countries. Each economic distribution displayed the average annual income of three population segments: the richest quintile of society, the middle quintile, and the poorest quintile. We varied the average income for each population segment to be either relatively low or high. Our goal was to measure how independent changes to the income held by each population segment influenced judgments of fairness and support for redistribution. The low income-level for each population segment is roughly equal to the actual average annual income for the richest, middle, and poorest quintiles of the United States population as of 2020. The high income-level was defined by simply doubling that of the low level. Specifically, we varied the economic distributions such that the richest quintile of society was described as having an average annual income of $220,000 or $440,000; the middle quintile was described as having an average income of $60,000 or $120,000; and the poorest quintile was described as earning and average of $14,000 or $28,000 (Table S4). Participants observed all combinations of income levels in a random order, which yielded a total of eight hypothetical economic distributions (i.e. two income levels for three population segments in a 2^3 within-subjects design). Participants were asked to assume that each distribution represented a Western, democratic country that was roughly equal in terms of population size, demographics, and total national wealth. While observing each distribution sequentially, participants rated the extent to which they thought each distribution was fair (-5 = completely unfair, +5 = completely fair) as well as their support for a government policy to redistribute wealth (-5 = strongly oppose, +5 = strongly support).

Table S5. Income Levels Used in Study 6
Study 6 stimuli included eight hypothetical income distributions using every combination of low and high income-levels for each population segment

**Population segment**	**Average Income: Low Level**	**Average Income: High Level**
**Richest 20% of households**	$220,000	$440,000
**Middle 20% of households**	$60,000	$120,000
**Poorest 20% of households**	$14,000	$28,000

After rating all eight combinations of average incomes, participants responded to a series of items asking their opinions on economic inequality. Participants rated the extent to which they agreed or disagreed with the following statements (-7=completely disagree, 0=neither agree nor disagree, 7=completely agree): “We ought to make sure that the POOREST members of society are doing as well as they can”; “We ought to build as strong, robust and wealthy of a MIDDLE class as possible”; and “We ought to make sure that it is possible to become EXTREMELY WEALTHY to incentivize people.” Lastly, participants completed the same set of questions on political attitudes as in previous studies.

7.2 Results. We created three indicator variables corresponding to whether participants were evaluating the low or high average income for each population segment (i.e., \$220,000 average income versus \$440,000 for the rich), clustering standard errors at the person-level. As predicted, doubling the average incomes of the richest and poorest population segments significantly predicted fairness ratings, whereas we observe no significant association between fairness ratings and the average income of the middle population segment (Table S5). Doubling the income of the poorest population segment led participants to rate economic distributions as significantly more fair. Doubling the income of the richest population segment led participants to rate economic distributions significantly less fair. Doubling the income of the middle population segment had no reliable effect on fairness ratings, however. This pattern of results is consistent with the SGI heuristic.

Table S6. Study 6 regression results predicting fairness ratings.

	Fairness Ratings			
	**b**	**se**	**t-val**	**p**
intercept	4.717	0.214	22.088	0.000***
doubling inc. of poor	1.199	0.125	9.613	0.000***
doubling inc. of middle	0.128	0.076	1.676	0.094
doubling inc. of rich	-0.730	0.101	-7.257	0.000***

Notes: *p<.05, **p<.01, ***p<0.001
Reporting unstandardized coefficients (and associated Standard Errors without corrections for multiple hypothesis testing, and two-sided t-tests) from an OLS model testing the effect of doubling the average income of the richest, middle, and poorest quintile on perceived fairness.

We find the same pattern of results when we control for participants’ opinions on economic inequality (Table S6). As expected, concern for the wellbeing of the poor has a main effect on fairness ratings. Furthermore, the effect of doubling the average income of the poor depends on

people's stated concern for the poor and, similarly, doubling the average income of the rich depends on concern for the rich. In contrast, we observe no interaction effect between doubling the average income of the middle class and stated concern for the middle class. This pattern of results suggests that people may not neglect the middle of economic distributions for principled reasons, but rather due the way in which people simplify information about economic distributions using the SGI heuristic.

Table S7. Study 6 regression results predicting fairness rating controlling for opinions on economic inequality.

	Fairness Ratings: Interactions with Concern			
	**beta**	**se**	**t-val**	**p**
intercept	8.158	0.994	8.208	0.000***
doubling inc. of poor	-0.802	0.364	-2.205	0.027*
concern for the poor	-0.359	0.072	-5.018	0.000***
doubling inc. of middle	-0.190	0.287	-0.663	0.507
concern for the middle	0.028	0.073	0.378	0.705
doubling inc. of rich	-1.366	0.194	-7.035	0.000***
concern for rich	0.082	0.046	1.801	0.072
doubling poor : concern poor	0.161	0.032	5.014	0.000***
doubling mid : concern mid	0.026	0.023	1.118	0.264
doubling rich : concern rich	0.076	0.021	3.589	0.000***

Notes: *p<.05, **p<.01, ***p<0.001
Reporting unstandardized coefficients (and associated Standard Errors without corrections for multiple hypothesis testing, and two-sided t-tests) from an OLS model testing the effect of doubling the average income of the richest, middle, and poorest quintile on perceived fairness.

We do not observe the same pattern of results with respect to support for redistribution (Tables S7 and S8). Only the income of richest segment of the population significantly predicts support for redistribution.

Table S8. Study regression results predicting support for redistribution.

	Support for Redistribution			
	**beta**	**se**	**t-val**	**p**
intercept	7.846	0.262	29.902	0.000***
doubling inc. of poor	-0.246	0.138	-1.784	0.074
doubling inc. of middle	-0.091	0.069	-1.333	0.182
doubling inc. of rich	0.376	0.084	4.446	0.000***

Notes: *p<.05, **p<.01, ***p<0.001
Reporting unstandardized coefficients (and associated Standard Errors without corrections for multiple hypothesis testing, and two-sided t-tests) from an OLS model testing the effect of doubling the average income of the richest, middle, and poorest quintile on support for redistribution.

Table S9. Study regression results predicting support for redistribution controlling for opinions on economic inequality: Interactions with Concern.

	Support for Redistribution			
	**beta**	**se**	**t-val**	**p**
intercept	2.230	1.087	2.052	0.040*
doubling inc. of poor	0.060	0.480	0.126	0.900
concern for the poor	0.403	0.084	4.822	0.000***
doubling inc. of middle	0.046	0.225	0.204	0.838
concern for the middle	0.169	0.087	1.951	0.051
doubling inc. of rich	0.459	0.157	2.915	0.004**
concern for wealth growth	-0.173	0.053	-3.242	0.001**
doubling poor : concern poor	-0.025	0.041	-0.603	0.547
doubling mid : concern mid	-0.011	0.019	-0.589	0.556
doubling rich : concern rich	-0.010	0.017	-0.587	0.557

Notes: *p<.05, **p<.01, ***p<0.001
Reporting unstandardized coefficients (and associated Standard Errors without corrections for multiple hypothesis testing, and two-sided t-tests) from an OLS model testing the effect of doubling the average income of the richest, middle, and poorest quintile on support for redistribution.

8. Study 7: Middle Neglect May be Caused by a Cognitive Blind Spot

Study 7 was designed to further test the claim that fairness evaluations are relatively less sensitive to changes in the welfare of the middle (compared to changes in welfare of the extremes) due to a cognitive blind spot (i.e., unintentional neglect) rather than intentional, principled disregard.

8.1 Methods and Participants. After excluding 33 participants according to our pre-registration, we were left with a sample of 868 participants from Amazon's Mechanical Turk who met pre-registered inclusion criteria (45% female, $M_{age} = 41.7$, $SD_{age} = 13.1$). Participants were shown sixteen hypothetical economic distributions—corresponding to all 2^4 combinations of a low and high value of wealth held by each of four wealth quartiles—with all values represented below.

Table S10. Summary of stimuli used in Study 7
These average income values were used to determine the four quartiles of income, either at the Low or High level.

	Low Income Level	High Income Level
**Richest**	$230,000	$460,000
**Upper-Middle**	$110,000	$220,000
**Lower-Middle**	$50,000	$100,000
**Poorest**	$20,000	$40,000

Notice that the high-income level is always double the low-income level for a given quartile, and the low-income level is always $10,000 more than the high-income level of the poorer quartile. We randomly assigned participants to one of three between-subjects conditions, depicted in Figure S4, in which we: (A) visually highlighted the wealthiest and least wealthy groups (i.e., the top and bottom rows in the table were highlighted in yellow); (B) visually highlighted the middle groups (i.e., the upper-middle and lower-middle groups); or (C) highlighted none of the rows. For each distribution shown, we asked participants to rate the fairness of that distribution

Figure S4. Stimuli used in Study 7, demonstrating the three highlighting conditions
(A). Top-Bottom Highlighting

	Average Annual Income
RICHEST 25% of households	$460,000
UPPER-MIDDLE 25% of households	$220,000
LOWER-MIDDLE 25% of households	$50,000
POOREST 25% of households	$20,000

(B) Highlighting the Middle

	Average Annual Income
RICHEST 25% of households	$230,000
UPPER-MIDDLE 25% of households	$110,000
LOWER-MIDDLE 25% of households	$100,000
POOREST 25% of households	$40,000

(C) No Highlighting

	Average Annual Income
**RICHEST** 25% of households	$230,000
**UPPER-MIDDLE** 25% of households	$110,000
**LOWER-MIDDLE** 25% of households	$50,000
**POOREST** 25% of households	$20,000

We pre-registered our hypothesis that highlighting the extremes of an economic distribution would make a smaller difference in sensitivity to changes of the values in the extremes (compared to no highlighting) than the comparatively larger effect of highlighting the *middle* groups on sensitivity to changes in the *middle* (compared to no highlighting). To test this, we estimated an OLS regression predicting fairness judgment from the fully saturated model corresponding to the interaction of: (1) the within-subject experimental factor of low-versus-high levels for all four wealth quartiles, and (2) two indicator variables corresponding to the between-subjects experimental condition of seeing the middle or extreme quartiles highlighted (hence the control condition served as the reference category). We furthermore controlled for stated concern for the economic well-being of each economic quartile. Standard errors were clustered at the individual-level. Analytically, our hypothesis comes to the prediction that: (a) the average of the regression coefficients of [poor_high_wealth*highlight_extremes & rich_high_wealth*highlight_extremes] *would be smaller than* (b) the average of the regressions coefficients of [lower-middle_high_wealth*highlight_middle & uppermiddle_high_wealth*highlight_middle].

8.2 Results. We estimated the linear combinations of these coefficients then bootstrapped 10,000 samples to estimate the mean difference of the foregoing comparison as well as the 95% confidence interval. As predicted, highlighting the extreme groups had a small effect on fairness judgements, whereas highlighting the middle groups had a much larger effect (b=.083, 95% CI [.05, .11]). We also tested a related comparison: (a) [how much more highlighting extremes increases sensitivity to the extremes (compared to no highlighting) than it increases sensitivity to the middle (compared to no highlighting)] VERSUS (b) [how much more highlighting the middle increases sensitivity to the middle (compared to no highlighting) than it increases sensitivity to the extremes (compared to no highlighting)]. The following figure compares the difference-in-differences between the pink and blue points for Extremes highlighted versus none compared to the difference-in-differences between the pink and blue points for Middle highlighted versus none. We find a similar estimated effect (b=0.093, 95% CI [0.05, 0.13]). These results are consistent with a “cognitive blind spot”, rather than ideological, explanation for the phenomenon of middle neglect.

Figure S5. Results of Study 7

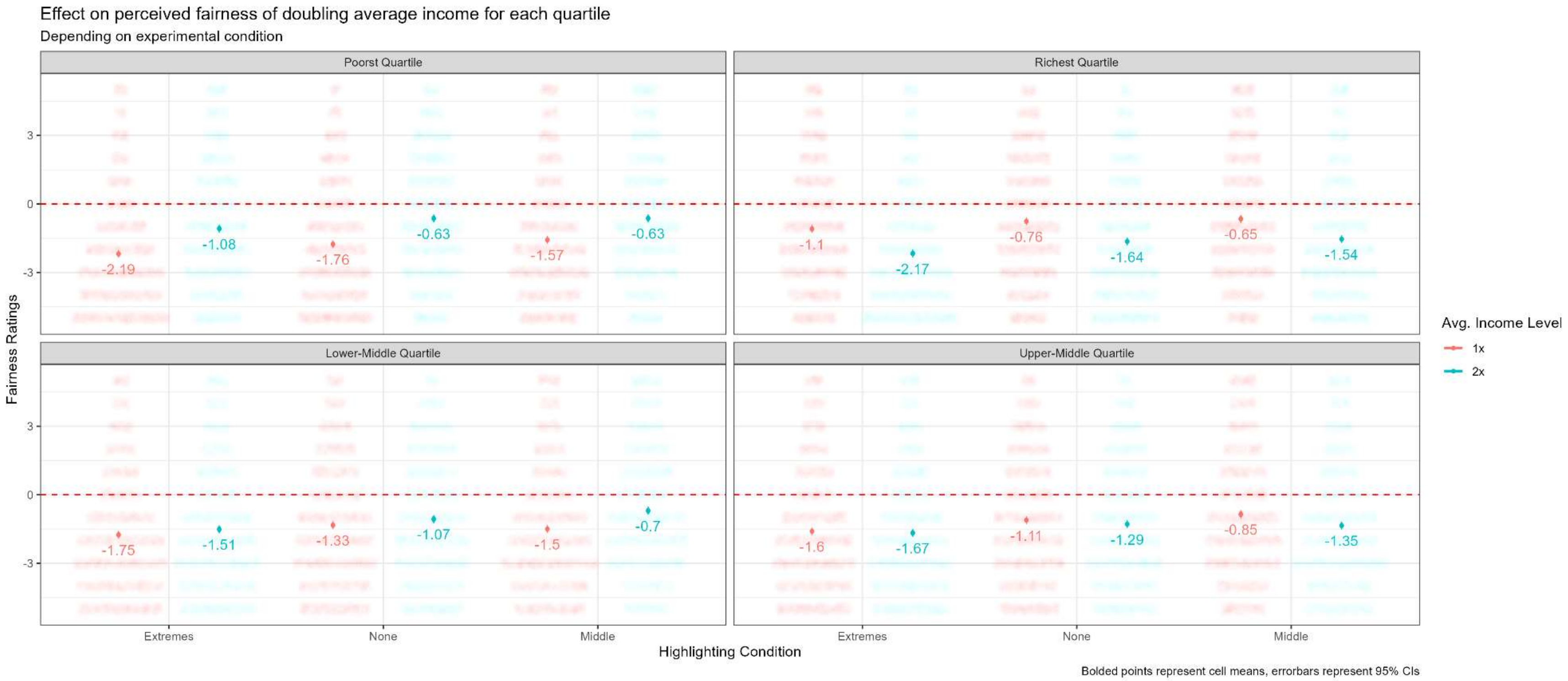

Note: Displaying means (dark points) and 95% Confidence Intervals for treatment effects of Highlighting Condition on Fairness ratings. Each translucent point represents one participant's response (*N*=868).

Table S11. Regression Results from Study 7

OLS regression results.

	Dependent variable:			
	Rating			
	Fairness	Fairness	Support Redist.	Support Redist.
	(1)	(2)	(3)	(4)
Poor Hi	0.512***<br>t = 20.019<br>p = 0.000	0.507***<br>t = 19.861<br>p = 0.000	−0.368***<br>t = −14.627<br>p = 0.000	−0.364***<br>t = −14.501<br>p = 0.000
Lower-m Hi	0.261***<br>t = 11.668<br>p = 0.000	0.262***<br>t = 11.608<br>p = 0.000	−0.116***<br>t = −6.684<br>p = 0.000	−0.116***<br>t = −6.604<br>p = 0.000
Upper-m Hi	−0.142***<br>t = −7.010<br>p = 0.000	−0.144***<br>t = −7.165<br>p = 0.000	0.070***<br>t = 4.050<br>p = 0.0001	0.074***<br>t = 4.354<br>p = 0.00002
Rich Hi	−0.491***<br>t = −22.789<br>p = 0.000	−0.487***<br>t = −22.617<br>p = 0.000	0.356***<br>t = 16.676<br>p = 0.000	0.352***<br>t = 16.539<br>p = 0.000
Highlight Extreme	−0.217*<br>t = −2.315<br>p = 0.021	−0.209**<br>t = −2.663<br>p = 0.008	0.175<br>t = 1.345<br>p = 0.179	0.165<br>t = 1.546<br>p = 0.123
Highlight Middle	0.050<br>t = 0.543<br>p = 0.587	0.014<br>t = 0.182<br>p = 0.856	0.044<br>t = 0.334<br>p = 0.739	0.096<br>t = 0.933<br>p = 0.351
Concern for Poor		−0.411***<br>t = −8.149<br>p = 0.000		0.561***<br>t = 8.063<br>p = 0.000
Concern for Lower-m		−0.033<br>t = −0.647<br>p = 0.518		0.138*<br>t = 2.010<br>p = 0.045
Concern for Upper-m		0.073**<br>t = 2.629<br>p = 0.009		−0.125***<br>t = −3.385<br>p = 0.001
Concern for Rich		0.196***<br>t = 7.724<br>p = 0.000		−0.296***<br>t = −9.118<br>p = 0.000
PoorHi X HghlghtExtreme	−0.006<br>t = −0.249<br>p = 0.804	−0.007<br>t = −0.262<br>p = 0.793	0.010<br>t = 0.400<br>p = 0.689	0.009<br>t = 0.359<br>p = 0.720
PoorHi X HghlghtMiddle	−0.048′<br>t = −1.869<br>p = 0.062	−0.052*<br>t = −2.050<br>p = 0.041	0.022<br>t = 0.839<br>p = 0.402	0.027<br>t = 1.062<br>p = 0.289
LowerMHi X HghlghtExtreme	−0.006<br>t = −0.342<br>p = 0.733	−0.007<br>t = −0.376<br>p = 0.707	0.020<br>t = 1.274<br>p = 0.203	0.020<br>t = 1.263<br>p = 0.207
LowerMHi X HghlghtMiddle	0.134***<br>t = 5.645<br>p = 0.00000	0.135***<br>t = 5.673<br>p = 0.000	−0.051**<br>t = −2.595<br>p = 0.010	−0.050*<br>t = −2.552<br>p = 0.011
UpperMHi X HghlghtExtreme	0.025′<br>t = 1.919<br>p = 0.056	0.027*<br>t = 2.044<br>p = 0.041	−0.037**<br>t = −3.107<br>p = 0.002	−0.036**<br>t = −3.087<br>p = 0.003
UpperMHi X HghlghtMiddle	−0.080***<br>t = −3.855<br>p = 0.0002	−0.084***<br>t = −4.048<br>p = 0.0001	0.035*<br>t = 2.024<br>p = 0.044	0.039*<br>t = 2.283<br>p = 0.023
RichHi X HghlghtExtreme	−0.047*<br>t = −2.076<br>p = 0.038	−0.046*<br>t = −2.003<br>p = 0.046	0.015<br>t = 0.722<br>p = 0.471	0.014<br>t = 0.664<br>p = 0.507
RichHi X HghlghtMiddle	−0.001<br>t = −0.028<br>p = 0.978	0.001<br>t = 0.055<br>p = 0.957	0.008<br>t = 0.374<br>p = 0.709	0.005<br>t = 0.241<br>p = 0.810
Constant	−1.365***<br>t = −14.709<br>p = 0.000	0.149<br>t = 1.009<br>p = 0.314	0.877***<br>t = 6.728<br>p = 0.000	−1.446***<br>t = −8.266<br>p = 0.000
Observations	13,888	13,760	13,888	13,760
R^2	0.084	0.278	0.026	0.322
Adjusted R^2	0.083	0.277	0.025	0.321
Residual Std. Error	2.667 (df = 13873)	2.370 (df = 13741)	3.399 (df = 13873)	2.840 (df = 13741)

Note: ′p<.1, *p<.05, **p<.01, ***p<.001

9. Study 8: Thought-Elicitation Procedure to Explore Middle Neglect

Study 8 further examines "middle neglect" using a thought-elicitation procedure adapted from Query Theory (Johnson, Häubl, and Keinan, 2007). This method allowed us to investigate which information commands people's attention when evaluating economic distributions.

9.1 Methods and Participants. After excluding 20 participants per our pre-registration, in Study 8 we recruited 269 participants from Amazon's Mechanical Turk who met pre-registered inclusion criteria (53% female, $M_{age} = 38.3$, $SD_{age} = 12.0$). Participants were asked to list the thoughts that occurred to them while observing the economic distributions for an anonymous country. To ensure that our results were not being driven by the particularities of a given distribution or the way it was presented, participants were randomly assigned to observe one of six possible distributions corresponding to a 2 (distribution: highly unequal versus highly equal) x 3 (presentation: table, pie chart, bar chart) between-subjects design. Participants evaluated the economic distribution on fairness and considered their support for redistribution, as in previous studies. Next, participants listed each of the thoughts that occurred to them as they made these evaluations. Participants could list up to seven thoughts as open-ended text (number of thoughts listed: $M_{thoughts} = 3.2$, $SD_{thoughts} = 1.4$). Lastly, participants self-coded each of their listed thoughts into one of the following seven categories:

1. I focused on how much of the total wealth was concentrated at the TOP of the distribution
2. I focused on how much of the total wealth was at the BOTTOM of the distribution
3. I focused on the DIFFERENCE between the amount of wealth at the TOP vs the BOTTOM of the distribution
4. I focused on the RATIO of wealth at TOP to wealth at the BOTTOM of the distribution
5. I focused on how much of the total wealth was held by the MIDDLE of the distribution
6. I focused on how much the distribution differed from total equality across groups
7. Other

If participants selected "other," they were asked to create their own label. We were primarily interested in the extent to which people focused on each part of the distribution.

9.2 Results. Using thought-elicitation data, we observed the extent to which people focused on each part of the distribution when evaluating an economic distribution. Participants focused on the top of the distribution, the bottom of the distribution, and a comparison of the top and bottom. In contrast, participants devoted relatively little attention to the middle of the distribution Figures S4 and S5). "Wealth held by the middle of the distribution" was the least commonly mentioned thought (52 instances).

Figure S6. Results of Study 8

Self-categorized areas of focus when evaluating of an economic distribution (*N*=269).

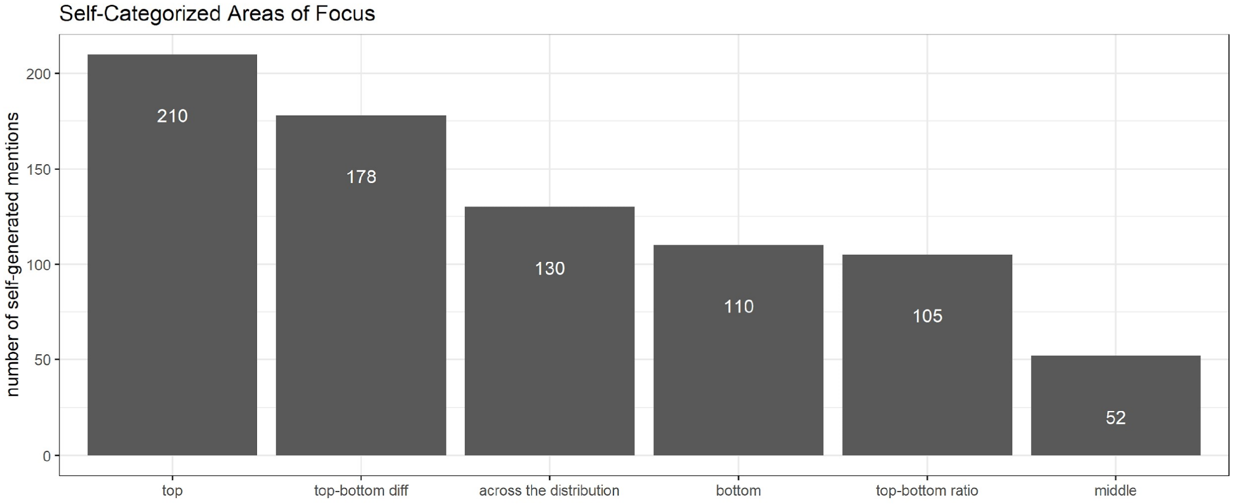

Figure S7. Study 8, Self-coded Responses

Self-categorized areas of focus when evaluating of an economic distribution, results by condition (*N*=269).

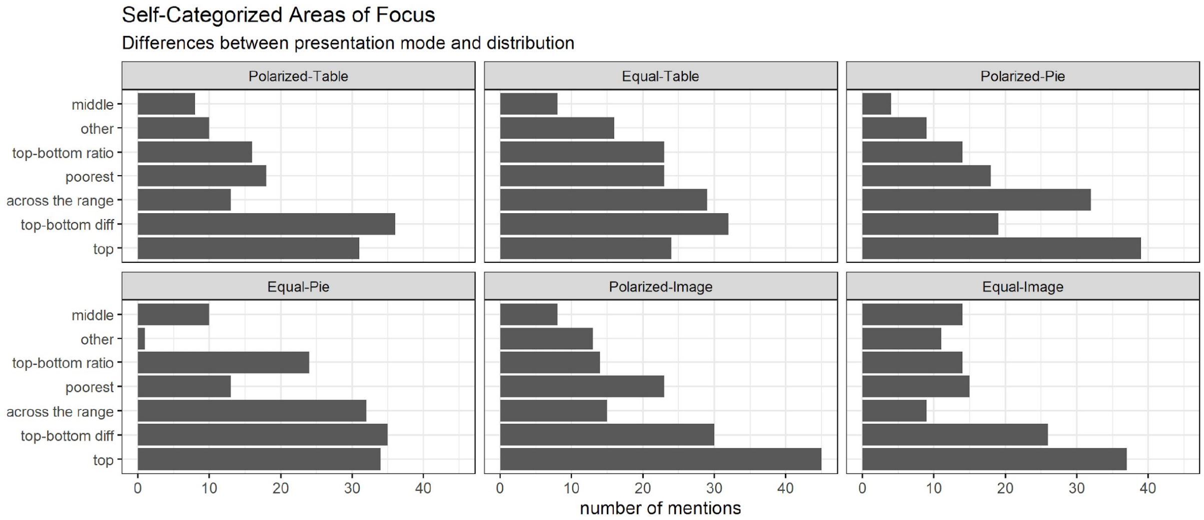

We found that people pay little attention to the middle of the economic distribution. We find further that this is true of both more and less extremely unequal allocations, and true across presentation formats. These results are consistent with the claim that disregard of the middle class is not ideologically motivated but rather a "cognitive blind spot" such that people tend to over-weight information about the extremes of a distribution relative to the middle.

10. Study 9: Mitigating Middle Neglect with Visual Displays

In Study 9, we tested a method to reduce middle neglect using visual displays of economic distributions, which facilitate a more holistic processing of information.

10.1 Methods and Participants. In Study 9, we recruited 400 participants from Amazon's Mechanical Turk, analyzing results of 393 participants who met pre-registered inclusion criteria (50% female, M_{age} = 39.2, SD_{age} = 11.6). Participants observed a pair of wealth distributions for anonymous countries with the populations partitioned into quartiles. One of these distributions had a larger gap between the richest and the poorest quartiles but a smaller gap between intermediate quartiles ("Middle Fair"). The other distribution had a smaller gap between the richest and poorest quartiles but a larger gap between the intermediate quartiles ("Top-Bottom Fair"). Participants were randomly assigned to observe the distributions presented either as tables or as bar graphs. While observing the pair of distributions, participants made a binary choice: "which of these two distributions seems to you to be more fair?"

We pre-registered a prediction that participants would be more likely to choose the Middle Fair distribution when these distributions were displayed as bar graphs since a visual display facilitated an evaluation of the full distribution.

10.2 Results. As predicted, participants were more likely to choose the Middle Fair distribution as the "more fair" alternative when they viewed the pair of distribution as bar graphs rather than as tables. These results are robust controlling for socioeconomic status, fiscal conservatism, and opinions on economic inequality (Table S8).

Table S12. Logistic regressions results from Study 8, predicting choices among distributions

	Dependent variable:			
	likelihood of choosing the Middle Fair distribution			
	(1)	(2)	(3)	(4)
graphical display	1.031***	1.078***	1.136***	1.125***
	(0.263)	(0.266)	(0.274)	(0.274)
	p<.001	p<.001	p<.001	p<.001
MacArthur SES		0.164*	0.107	0.069
		(0.080)	(0.085)	(0.090)
		p=.040	p=.208	p=.443
fiscal conservatism			0.340***	0.257**
			(0.077)	(0.099)
			p<.001	p=.0096
WVS: Incentivize				0.058
				(0.064)
				p=.366
WVS: Success as Luck				-0.049
				(0.060)
				p=.414
Constant	-1.385***	-2.166***	-3.302***	-2.785***
	(0.131)	(0.411)	(0.521)	(0.761)
	p<.001	p<.001	p<.001	p<.001
Observations	393	393	393	393
Log Likelihood	-194.402	-192.250	-181.642	-180.736
Akaike Inf. Crit.	392.803	390.501	371.284	373.471

Note: *p<0.05, **p<0.01, ***p<0.001

Here we report unstandardized coefficients along with standard errors from OLS regression models (two-sided tests, no multiple-hypothesis corrections).

These results are consistent with the hypothesis that people afford greater weight to the middle of economic distributions when information is displayed graphically rather than tabularly. When people view economic distributions tabularly, they have a harder time representing the full set of information and are instead more likely to rely on the SGI heuristic. People wishing to convey information about inequality would do well to present this information graphically thereby facilitating a more even-handed evaluation of information across the distribution.

11. Auditing Popular Representations of Inequality

We sought to measure how well the popular press represents economic inequality. Specifically, we wanted to audit the frequency with which newspapers present information about economic inequality in a way that facilitates unbiased interpretation of the data. Thus, a trained team of Research Assistants (RAs) read every relevant article in the year 2020 from a set of newspapers, coding them according to various features.

11.1 Methods

We first constructed a database of every relevant article from the year 2020 printed in one of the eight most popular newspapers (by circulation[1]): New York Times, USA Today, Tampa Bay Times, Washington Post, Wall Street Journal, Los Angeles Times, Chicago Tribune, Houston Chronicle. We searched Factiva, an online database including those periodicals, and collected every article title containing one of the following search terms:[2]

- Income inequality
- Wealth inequality
- Income equality
- Wealth equality
- Income distribution
- Wealth distribution
- Income allocation
- Wealth allocation
- Inequality

This returned 3128 unique articles. After several rounds of training, a team of RAs read through the results of this query and selected all articles relevant for our purposes—those with a visual, tabular, or verbal representation of how money is distributed among people. This resulted in a collection of 58 articles with visual displays of economic distributions and 335 articles with verbal descriptions of economic distributions. Of these, RAs then evaluated each distribution representation along the following dimensions:

---

[1] https://www.agilitypr.com/resources/top-media-outlets/top-15-daily-american-newspapers/

[2] All access was obtained, and permissions granted, per the license with UCLA Libraries.

- Visual
  - Graph type
  - Time series
  - Unequal bin sizes (i.e., comparing richest 10% versus rest of country)
  - Number of bins represented
  - Whether population categories are exclusive
  - Whether population categories are exhaustive
  - X-axis & Y-axis metrics
- Verbal
  - Unequal bin sizes
  - Scaled (e.g., average income) or aggregate (e.g., total share of wealth held) metric
  - Explicit mention of intermediate income group

One lead RA, who helped to develop this coding scheme, arbitrated any disagreement or confusion about the way that an article ought to be evaluated along one of the dimensions.

11.2 Results

Table S13. Results of Newspaper Audit

Results of the audit of popular representations of economic distributions.	
**Visual Depictions (58 graphs)**	
Unequal Bin Sizes	77%
**Verbal Depictions (335 graphs)**	
Unequal Bin Sizes	94%
Aggregate Metric	62%
Unequal Bins with Aggregate Metric	63%
Explicit Mention of Extremes, Not Middle	57%

As can be seen, the majority of articles represent the distribution of money in ways that enable biased interpretation. It is no surprise, then, that so many people have such inaccurate views of inequality: Indeed, the most common ways of encountering information about inequality are opposed to even-handed evaluation of the data.

Finally, below (Table S14) we present the results of two tests of regression assumptions: (a) constant variance (i.e., homoskedasticity) between conditions using either Levene's test of homogeneity of variance between groups or (when pre-registered continuous controls were

included) the Score Test of non-constant variance, and (b) normality of residuals using a Shapiro-Wilks test of the distribution of variables. Unless otherwise noted, no adjustments were made in light of these test results.

Table S14. Test of regression assumptions

S#	Description	Test of Constant Variance	Test of Normality
**1**	*Partition Dependence: Scaled versus Unscaled Metric*	**Levene's Test:** $F(1, 389) = 4.85$, $p = 0.03$	**Shapiro-Wilks Test:** $W = 0.9076$, $p < .001$
**2**	*Partition Dependence: Quintiles versus Quasi-Logarithmic*	**Score Test:** $\chi^2(1) = 1.64$, $p = 0.20$	**Shapiro-Wilks Test:** $W = 0.92436$, $p < .001$
**3**	*Partition Dependence: {50-50} versus {10-10-10-10-10-50}*	**Levene's Test:** $F(242) = 4.94$, $p = .03$	**Shapiro-Wilks Test:** $W = 0.96$, $p = 0.005$
**4**	*Partition Dependence: Current wealth distribution given*	**Score Test:** $\chi^2(1) = 0.68$, $p = 0.41$	**Shapiro-Wilks Test:** $W = 0.91$, $p < .001$
**5**	*Partition Dependence: Population grouping x Metric*	**Levene's Test:** $F(3, 398) = 1.64$, $p = 0.18$	**Shapiro-Wilks Test:** $W = 0.93$, $p < .001$
**6**	*Middle Neglect: Low vs. High Average Income Tertiles*	**Score Test:** $\chi^2(1) = 0.57$, $p = 0.45$	**Shapiro-Wilks Test:** $W = 0.96$, $p < .001$
**9**	*Middle Neglect: Attenuation with Visual Displays*	**Levene's Test:** $F(1, 391) = 16.72$, $p < 0.001$	**Shapiro-Wilks Test:** $W = 0.62$, $p < .001$

This table reports the results of the tests of regression assumptions for each study presented in the manuscript. Homoskedasticity was tested using Levene's test of homogeneity of variance between groups or a Score Test of non-constant variance, and normality was tested using a Shapiro-Wilks test of the distribution of residuals.

Note that we did not test regression assumptions in Study 7 since point estimates and confidence intervals were obtained via bootstrapping. In Study 8, we also do not report assumptions tests since we offer no inferential statistical tests.
