# Supplementary material for: How common depictions of wealth distributions can bias people to underestimate inequality
Source: Nat Commun. 2026 Mar 6;17:3897. doi: 10.1038/s41467-025-62422-5 (PMC13125609; doi:10.1038/s41467-025-62422-5)
Supplement: Supplementary file 2 — Reporting Summary [file 41467_2025_62422_MOESM2_ESM.pdf]

nature portfolio

Corresponding author(s): Jonathan Bogard

Last updated by author(s): Dec 22, 2024

Reporting Summary

Nature Portfolio wishes to improve the reproducibility of the work that we publish. This form provides structure for consistency and transparency in reporting. For further information on Nature Portfolio policies, see our Editorial Policies and the Editorial Policy Checklist.

Statistics

For all statistical analyses, confirm that the following items are present in the figure legend, table legend, main text, or Methods section.

n/a	Confirmed	
☐	☒	The exact sample size (*n*) for each experimental group/condition, given as a discrete number and unit of measurement
☐	☒	A statement on whether measurements were taken from distinct samples or whether the same sample was measured repeatedly
☐	☒	The statistical test(s) used AND whether they are one- or two-sided<br>*Only common tests should be described solely by name; describe more complex techniques in the Methods section.*
☐	☒	A description of all covariates tested
☐	☒	A description of any assumptions or corrections, such as tests of normality and adjustment for multiple comparisons
☐	☒	A full description of the statistical parameters including central tendency (e.g. means) or other basic estimates (e.g. regression coefficient) AND variation (e.g. standard deviation) or associated estimates of uncertainty (e.g. confidence intervals)
☐	☒	For null hypothesis testing, the test statistic (e.g. *F*, *t*, *r*) with confidence intervals, effect sizes, degrees of freedom and *P* value noted<br>*Give P values as exact values whenever suitable.*
☒	☐	For Bayesian analysis, information on the choice of priors and Markov chain Monte Carlo settings
☒	☐	For hierarchical and complex designs, identification of the appropriate level for tests and full reporting of outcomes
☒	☐	Estimates of effect sizes (e.g. Cohen's *d*, Pearson's *r*), indicating how they were calculated

Our web collection on statistics for biologists contains articles on many of the points above.

Software and code

Policy information about availability of computer code

Data collection	All data were collected via Qualtrics surveys
Data analysis	All data were analyzed using R ('tidyverse' package)

For manuscripts utilizing custom algorithms or software that are central to the research but not yet described in published literature, software must be made available to editors and reviewers. We strongly encourage code deposition in a community repository (e.g. GitHub). See the Nature Portfolio guidelines for submitting code & software for further information.

Data

Policy information about availability of data

All manuscripts must include a data availability statement. This statement should provide the following information, where applicable:

- Accession codes, unique identifiers, or web links for publicly available datasets
- A description of any restrictions on data availability
- For clinical datasets or third party data, please ensure that the statement adheres to our policy

All data for the experiments have been anonymized and in are publicly available on ResearchBox: https://researchbox.org/380

Research involving human participants, their data, or biological material

Policy information about studies with human participants or human data. See also policy information about sex, gender (identity/presentation), and sexual orientation and race, ethnicity and racism.

Reporting on sex and gender	We report the percentage of each sample that is female, with the complement comprised of self-reported males, people who are non-binary, and people who opted to not report.
Reporting on race, ethnicity, or other socially relevant groupings	We did not collect racial demographic information, except when reported in the online supplement.
Population characteristics	See above.
Recruitment	Participants were collected using CloudResearch as a recruitment platform of Amazon's Mechanical Turk participants who had not participated in previous studies related to the project.
Ethics oversight	UCLA Institutional Review Board

Note that full information on the approval of the study protocol must also be provided in the manuscript.

Field-specific reporting

Please select the one below that is the best fit for your research. If you are not sure, read the appropriate sections before making your selection.

☐ Life sciences ☒ Behavioural & social sciences ☐ Ecological, evolutionary & environmental sciences

For a reference copy of the document with all sections, see nature.com/documents/nr-reporting-summary-flat.pdf

Behavioural & social sciences study design

All studies must disclose on these points even when the disclosure is negative.

Study description	Quantitative studies, often involving randomization
Research sample	Online participants from Amazon's Mechanical Turk, recruited via CloudResearch with approval rates >=95%, and who passed all pre-registered attention checks contained in the survey. Total N=3599 (approx. 49% Female, 38 years old).
Sampling strategy	Convenience sampling without any weighting or regard for participant demographics during recruitment. Randomization occurred inside of the survey using Qualtrics' native randomization tool. Non-representative sampling is appropriate considering the basic psychology involved, not expected to be importantly moderated by demographic characteristics. Sample sizes were estimated using back-of-the-envelope estimates of effect sizes and comparing against similar studies from other papers.
Data collection	All surveys were administered online, with subjects participating from their own devices at the location of their choosing. Data collection ranged from March, 2018 through October, 2023.
Timing	Participants were given approximately 10x the estimated time required to complete each survey. 20180301 20231003
Data exclusions	Data exclusions, as noted in all pre-registrations, were based on attention-based screeners: S1 (9 Ps), S2 (35 Ps), S3 (88 Ps), S4 (32 Ps), S5 (121 Ps), S6 (3 Ps), S7 (33 Ps), S8 (20 Ps), and S9 (7 Ps). We only capture data of those participants who consented and completed the entire study, responding to all measures.
Non-participation	Failure rates are reported in the supplement, usually around 10% of samples.
Randomization	When relevant, randomization occurred within the surveys using Qualtrics' randomization feature after consenting to participate.

Reporting for specific materials, systems and methods

We require information from authors about some types of materials, experimental systems and methods used in many studies. Here, indicate whether each material, system or method listed is relevant to your study. If you are not sure if a list item applies to your research, read the appropriate section before selecting a response.

Materials & experimental systems

n/a	Involved in the study
☒	☐ Antibodies
☒	☐ Eukaryotic cell lines
☒	☐ Palaeontology and archaeology
☒	☐ Animals and other organisms
☒	☐ Clinical data
☒	☐ Dual use research of concern
☒	☐ Plants

Methods

n/a	Involved in the study
☒	☐ ChIP-seq
☒	☐ Flow cytometry
☒	☐ MRI-based neuroimaging
